# Isoalantolactone inhibits pancreatic cancer proliferation by regulation of PI3K and Wnt signal pathway

**DOI:** 10.1371/journal.pone.0247752

**Published:** 2021-03-04

**Authors:** Chaoxiong Zhang, Lei Huang, Jingyuan Xiong, Linshen Xie, Shi Ying, You Jia, Yuqin Yao, Xuejiao Song, Zhenguo Zeng, Jialing Yuan

**Affiliations:** 1 Research Center for Occupational Respiratory Disease, West China Fourth Hospital, Sichuan University, Chengdu, China; 2 Healthy Food Evaluation Center, West China School of Public Health, Sichuan University, Chengdu, China; 3 Department of Medicine, University of Illinois College of Medicine, Chicago, Illinois, United States of America; 4 Department of Gastroenterology, Chengdu First People’s Hospital, Chengdu, China; 5 Department of Critical Care Medicine, The First Affiliated Hospital of Nanchang University, Nanchang, China; 6 Department of Obstetrics and Gynecology, West China Second University Hospital, Sichuan University, Chengdu, Sichuan, China; 7 Key Laboratory of Birth Defects and Related Diseases of Women and Children (Sichuan University), Ministry of Education, Chengdu, China; Chung Shan Medical University, TAIWAN

## Abstract

**Background/aims:**

Isoalantolactone (IATL) is one of multiple isomeric sesquiterpene lactones and is isolated from inula helenium. IATL has multiple functions such as antibacterial, antihelminthic and antiproliferative activities. IATL also inhibits pancreatic cancer proliferation and induces apoptosis by increasing ROS production. However, the detailed mechanism of IATL-mediated pancreatic cancer apoptosis remains largely unknown.

**Methods:**

In current study, pancreatic carcinoma cell lines (PANC-1, AsPC-1, BxPC-3) and a mouse xenograft model were used to determine the mechanism of IATL-mediated toxic effects.

**Results:**

IATL (20μM) inhibited pancreatic adenocarcinoma cell lines proliferation in a time-dependent way; while scratch assay showed that IATL significantly inhibited PANC-1 scratch closure (P<0.05); Invasion assays indicated that IATL significantly attenuated pancreatic adenocarcinoma cell lines invasion on matrigel. Signal analysis showed that IATL inhibited pancreatic adenocarcinoma cell proliferation by blocking EGF-PI3K-Skp2-Akt signal axis. Moreover, IATL induced pancreatic adenocarcinoma cell apoptosis by increasing cytosolic Caspase3 and Box expression. This apoptosis was mediated by inhibition of canonical wnt signal pathway. Finally, xenograft studies showed that IATL also significantly inhibited pancreatic adenocarcinoma cell proliferation and induced pancreatic adenocarcinoma cell apoptosis *in vivo*.

**Conclusions:**

IATL inhibits pancreatic cancer proliferation and induces apoptosis on cellular and *in vivo* models. Signal pathway studies reveal that EGF-PI3K-Skp2-Akt signal axis and canonical wnt pathway are involved in IATL-mediated cellular proliferation inhibition and apoptosis. These studies indicate that IATL may provide a future potential therapy for pancreatic cancer.

## Introduction

Pancreatic cancer is a pandemic disease and has increased all over the world in the past decades. Pancreatic cancer has been classified into exocrine and endocrine groups. The exocrine group is the majority (95%), in which ductal adenocarcinoma is the most common type. The endocrine group is characterized by hormone secretion, such as insulin, gastrin and glucagon from neuroendocrine tumor cells [1]. Pancreatic cancer is associated with a high death rate, poor outcome and rapid developments to later stages [2].

Pancreatic cancer is believed to be related to various epidemic factors, such as obesity, heavy alcoholic consumption, genetic causes, etc [3]. The tumorigenesis processes of pancreatic cancer may involve many signal pathways, such as TGFβ, PI3K-Akt, Wnt/β-Catenin, ROS, and cyclin-Dependent Kinase related signal pathways [4–7]. Recent studies indicate that not only genetic mutations, but also epigenetic modifications are involved in pancreatic tumorigenesis [8, 9].

Based on these signaling pathways, many novel therapeutic targets of pancreatic cancer have been developed. Current adjuvant chemotherapies include gemcitabine or 5-FU [10] while a combination chemotherapy such as FOLFIRINOX (folinic acid plus fluorouracil) has been found more effective than gemcitabine but has more side-effects [11]. Moreover, surgery with radiotherapy is also an option for pancreatic cancer patients [12]. Although more new medicines are being developed for clinical use, an ideal therapy does not yet exist for treating pancreatic cancer.

Sesquiterpene lactones are a group of chemicals containing lactone ring and normally exist in plants of asteraceae such as daisies and asters. Sesquiterpene lactones can be divided in several main classes: germacranolides, heliangolides, guaianolides, pseudoguaianolides, hypocretenolides and eudesmanolides. Isoalantolactone (IATL) is one of the isomeric sesquiterpene lactones isolated from inula helenium, which has shown multiple functions as an antibacterial, antihelminthic and antiproliferative agent [13]. IATL has also been reported to induce apoptosis in a pancreatic cancer cellular model by increasing ROS [14]. A recent study confirmed the anticancer effect of IATL by using combinations of alloalantolactone, alantolactone and IATL [15]. However, the detailed mechanism of IATL remains largely unknown.

In the current study, we used *in vitro* cellular and *in vivo* xenograft models to determine the effect of IATL on the fate of pancreatic cancer. Our study shows that IATL induces pancreatic cancer cell apoptosis by inhibition of the Wnt-β-catenin signal pathway. Furthermore, IATL inhibits pancreatic cancer cell proliferation by AMPK- Skp2-Akt signal pathway. Most importantly, the xenograft model shows that IATL inhibits pancreatic carcinoma cell growth and induces apoptosis *in vivo*.

## Material and methods

### Cell culture and IATL

Human pancreatic ductal adenocarcinoma (PDAC) cell lines (PANC-1, AsPC-1 and BxPC-3) were purchased from American Type culture collection (ATCC, Manassas, VA). All cell lines used in this study were authenticated and characterized, and the mycoplasma testing of these cell lines are negative. The cells were cultured in Dulbecco’s Modified Eagle’s Medium (DMEM) supplemented with 10% fetal bovine serum containing 100 units/mil penicillin and streptomycin. The cells were incubated at 37°C in 5%CO2 and were used at passages 5–20. All experiments and measurements were carried out in accordance with the relevant guidelines and regulations of the universities. IATL were purchased from Sigma-Aldrich (PHL89228, St. Louis, MO. USA).

### Cell viability assay (MTT)

PANC-1, AsPC-1 and BxPC-3 cells were seeded in 96 wells plate (10^4^/ well). The following day, the cells were treated with 5% DMSO or IATL for 72 hours. Cell proliferation was determined by MTT assay kit (Sigma, St. Louis, MO). The total live cells were also stained by trypan blue and then counted by TC 10 automated cell counter (Bio-Rad Inc., Hercules, CA) [16].

### Wound closure assay and invasion assay

PANC-1 Cells were seeded in 6 wells plate and scratched by a sterilized 200μl pipette tip. After that, cells were treated with DMSO or IATL for 24 hours. Phase contrast images were taken at beginning and after 24 hours and 48 hours. The cell migration effects were measured by the closure area of scratch [17]. Invasion assays were performed on an 8μm pore size transwell (BD Falcon cell culture insert, BD Bioscience) with modifications. Invasion assays were performed in 24 well transwell inserts which were coated with 100 μL matrigel, 1×10^5^ PANC-1, AsPC-1 and BxPC-3 cells were transfected with pLV-GFP virus (vectorbuilder, Chicago, Illinois). After 3 days incubation, more than 90% of each of the three cell lines were GFP-positive. The cells then were seeded into the insert. The cells were cultured in 10% FBS complete DMEM medium in presence or absence of ITAL. The bottom chambers contained 0.5ml 10% FBS complete DMEM medium. After 24 hours, the cells in the inserts were removed using a cotton swab. The cells on the bottom side of insert were covered by prolong Gold Antifade mountant with coverslides (Therm Fisher Scientific, Waltham, MA). The GFP positive cells were counted using a Nikon fluorescent microscope (Nikon, Eclipse TE2000-S, Nikon Instrument Inc, Melville, NY) [18].

### Xenograft models

A total 36 BALB/c nude male mice (4–5 weeks old) were purchased and were divided into 6 groups (Beijing HFK Bioscience Co., Ltd., Beijing, China). The six groups included PANC-1, PANC-1/ IATL; AsPC-1, AsPC-1/ IATL; BxPC-3, BxPc-3/IATL treatment groups. All animal experiments were reviewed and approved by the Institutional Animal Care and Use committee at Sichuan University (protocol number: 20190089). The BALB/c nude male mice were kept in mouse cage (CAVENS, Changzhou, China) located in the mouse room with 24°C temperature, 50% humidity, the food for mice were purchased from CAVENS, the autoclaved drinking water were provided. Nestlets were provided for environmental enrichment. Once the mice were anesthetized by ketamine and xylazine (The used dose of ketamine/xylazine for this experiment 100 mg/kg and 10 mg/kg body weight. Here 1.6mg ketamine and 0.16mg xylazine were diluted in 100 μl PBS for intraperitoneal injection), PANC-1, AsPC-1 or BxPC-3 cells (1x10^6^ cells in 100μl PBS) were injected into left flank of the mice subcutaneously. Mice were kept warm using heat lamp and be checked frequently 15 min until wake. For the health of mice, these mice were checked and food and water intake were monitored daily. IATL or PBS were given to the mice (0.5mg/kg, IP) once a week for 5 weeks. The tumor sizes were recorded every week. We believed that large volume tumor may be a better indictor to reflect the effect of IATL on tumor growth. Institutional Animal Care and Use Committee at Sichuan University specifically reviewed and believed that large tumor volumes do not bring additional and unnecessary discomfort to these mice. This Committee approved the use of large tumor volumes this study. The largest (a) and smallest diameters (b) of each xenograft were measured twice. Volume (V) was estimated using the formula V = 0.52×a^2^×b. The mean tumor volume were recorded as tumor growth curves for each group of mice. Both tumor weights and volume were also measured after the mice were euthanized by anesthesia of CO2 and dislocation of cervical spine at the end of experiment. The tumor growth was evaluated as the mean volume± SE. At the end of experiment, the IATL-mediated apoptosis in tumors was evaluated using flow cytometry [19, 20].

### Apoptosis flow cytometry assay

The xeonograft cell apoptosis was also evaluated by flow cytometric detection of phosphotidyl serine externalization using the FITC annexin V apoptosis detection kit I (cat.no. 556547, BD Biosciences, San Jose, Ca). After the mice were treated with vehicle or IATL, xenografts were minced and washed once with cold PBS. The cells were then digested with collagenase and dispase for 30min at 37°C. The suspensions were filtered using a cell strainer (70μm). The filtered solution was centrifuged at 300g for 10min. The pellet was washed with PBS once. The cell pellets were resuspended in 1×binding buffer and incubated with 5μl FITC annexin V and 5 μl PI solution. After incubation for 15 minutes at room temperature, binding buffer was added. FITC annexin V and propidium iodide (PI) positive cells were determined by FACS caliber flow cytometry (BD Bioscience, San Jose, CA) [21].

### Western blotting

Samples were harvested with RIPA buffer (cat.no. ab15634, Abcam) containing proteinase inhibitors and phosphatase inhibitors as per standard protocols. After sonication and centrifugation, the supernatant was collected, Laemmli sample buffer was added. And then the samples were boiled and subsequently analyzed by SDS–PAGE. After transfer to a nitrocellulose membrane (Bio-Rad, Inc., Hercules, CA), Western blotting was performed using appropriate primary antibodies and horseradish peroxidase-conjugated secondary antibodies prior to visualization via chemiluminescence (Amersham Biosciences, Piscataway, NJ). The used antibodies were listed as followed: Anti-AMPK (phosphor T183+T172) antibody (1:1500, cat.no. ab133448, Abcam, Cambridge, UK), anti-AMPK alpha 1 antibody (1:2000, cat.no. ab133448, Abcam), the customed phosphor-Skp2 S256 antibody is from Jiaxing Xinda Biotechnology Co. Ltd (1:1000), anti-Skp2 antbody (1:1000, cat.no. ab19877, Abcam), anti-AKT antibody (1:1000, cat.no. ab179463, Abcam), anti-AKT1 (phosphor S473) antibody (1:1000, cat.no. ab81283, Abcam), anti-caspase-3 antibody (1:2000, cat.no. ab813847, Abcam), anti-bax antibody (1:1000, cat.no. ab32503, Abcam), anti-phospho-GSK-3β (Ser9) antibody (1:1000, cat.no. #9336, Cell Signaling TECHNOLOGY, Danvers, Massachuset), anti-GSK-3β antibody (1:1000, cat.no. #9315, Cell Signaling TECHNOLOGY,), anti-β catenin antibody (1:1000, cat.no. 16051, Abcam), anti-β catenin phosphor Y142 antibody (1:1000, cat.no. 27798, Abcam), anti-Lamin B1 antibody (1:1000, cat.no.16048, Abcam). Anti-β-actin antibody (1:2000, cat.no.8227, Abcam). Blot density was determined by Alpha Imager software (Alpha Innotech, San Leandro, CA) [22].

### Statistical analysis

All data were analyzed using SPSS software version 16.0 (SPSS, Inc.) Student’s t-test was used to compare the means of data from two experimental groups while significant differences (p < 0.05) amongst multiple group comparisons were confirmed by one-way ANOVA and Tukey’s post hoc multiple comparisons testing. Results are expressed as means ± SE [22].

## Results

### IATL inhibited pancreatic adenocarcinoma cell proliferation

**Fig 1:**
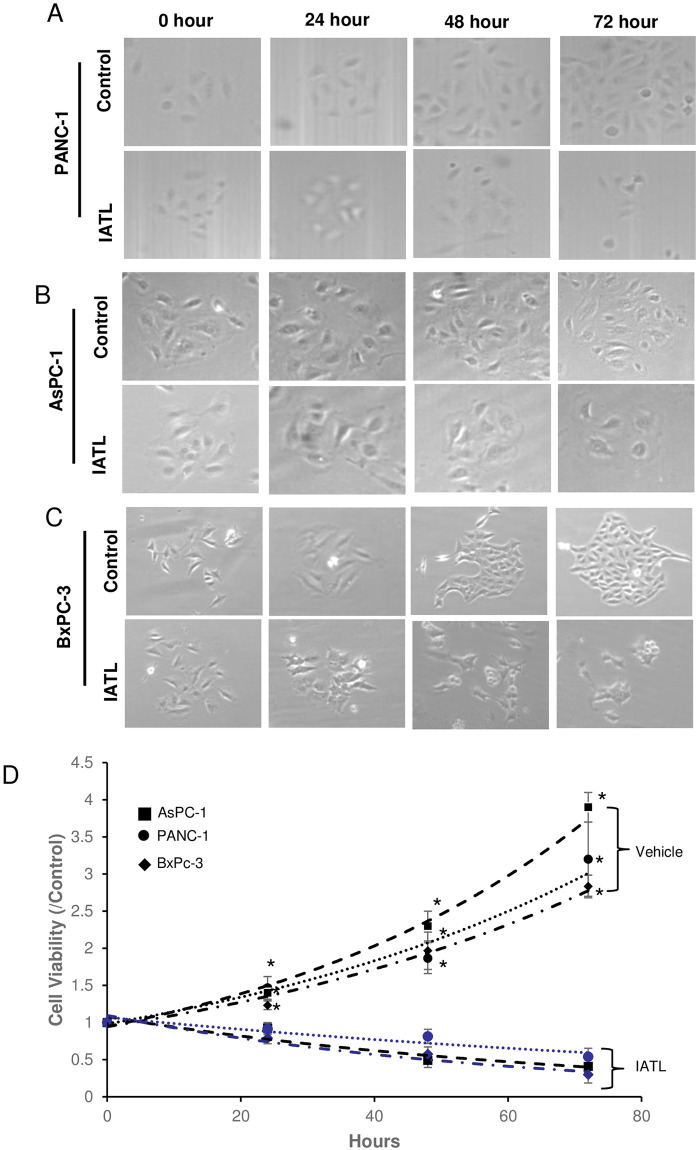
IATL inhibits pancreatic cancer cell proliferation. The PANC-1, AsPC-1 or BxPC-3 cells were treated with 20μM IATL at the indicated times. After the cells were digested with trypsin, the cellular viability was determined by MTT assay. (A) representative images of PANC-1 were treated with IATL; (B) The morphological characteristic changes in AsPC-1 when treated with IATL; (C) The morphological characteristic changes in BxPc-3 when treated with IATL; (D) MTT assay indicated that IATL significantly inhibited cell viability in each cell line (*P<0.05, n = 4).

IATL has been reported to induce apoptosis of pancreatic carcinoma in both cellular and *in vivo* models. The underlying mechanism of IATL-mediated apoptosis has been reported as a ROS-induced toxic effect [14]. However, ROS-induced pancreatic carcinoma apoptosis did not account for these complicated processes. In this study, three different pancreatic cancer cell lines, PANC-1, AsPC-1 and BxPC-3 were cultured in 96 wells plates in the presence of 20μM IATL and proliferation was tested at various time. The colony and cell count showed that IATL significantly lowered both colonies (Fig 1A–1C, Table 1) and total live cell numbers compared to control (Table 2). The MTT assay showed that IATL time-dependently inhibited PANC-1s proliferation after 24 hours treatment (Fig 1D; P<0.05). IATL also showed a significant inhibitory effect on cell proliferation on both AsPC-1 and BxPC-3 after 24 hours treatment (Fig 1D; P<0.01).

**Table 1:** IATL inhibited pancreatic carcinoma cells colony development.

Time (Hrs)	PANC-1		AsPC-1		BxPC-3	
	Vehicle	IATL	Vehicle	IATL	Vehicle	IATL
**0**	14.7±0.57	15.0±1.0	15.7±1.15	15.0±1.0	15.3±1.52	16.0±1.0
**24**	17.6±0.53	16.7±0.58	19.7±1.58	16.3±1.15	19.7±2.52	17.7±1.52
**48**	24.0± 1.0	9.0±1.0*	25.3±1.52	12.6±0.52*	22.6±2.08	11.0±1.0*
**72**	31.3±1.5	7.6±0.57*	32.3±2.12	9.67±1.52*	36.3±2.51	7.33±0.57*

The pancreatic carcinoma cells were seeded into 12 wells plate. The colony number was counted under microscope. Each well was counted 5 independent visual fields. The number of colonies was represented as mean±SE.

Colony number/visual field (* P<0.05).

**Table 2:** IATL inhibited pancreatic carcinoma cells proliferation.

Time (Hrs)	PANC-1		AsPC-1		BxPC-3	
	Vehicle	IATL	Vehicle	IATL	Vehicle	IATL
**0**	1.0±0.10	1.0±0.1	1.0±0.15	1.0±0.10	1.0±0.1	1.0±0.1
**24**	1.30±0.10	1.41±0.1	1.6±0.1	1.43±0.15	1.4±0.10	1.1±0.26
**48**	2.96±0.25	0.8±0.1*	3.26±0.21	0.56±0.11*	3.26±0.21	0.56±0.06*
**72**	4.30±0.26	0.6±0.1*	5.13±0.21	0.43±0.05*	3.80±0.1	0.38±0.05*

The pancreatic carcinoma cells were seeded into 12 wells plate and were collected at indicated time. After the cells centrifuged, the cells were resuspended in 1ml PBS and were counted by TC 10 automated cell counter.

Trypan Blue staining negative cell (×105/mL, * P<0.05).

### IATL inhibited pancreatic adenocarcinoma cell migration

**Fig 2:**
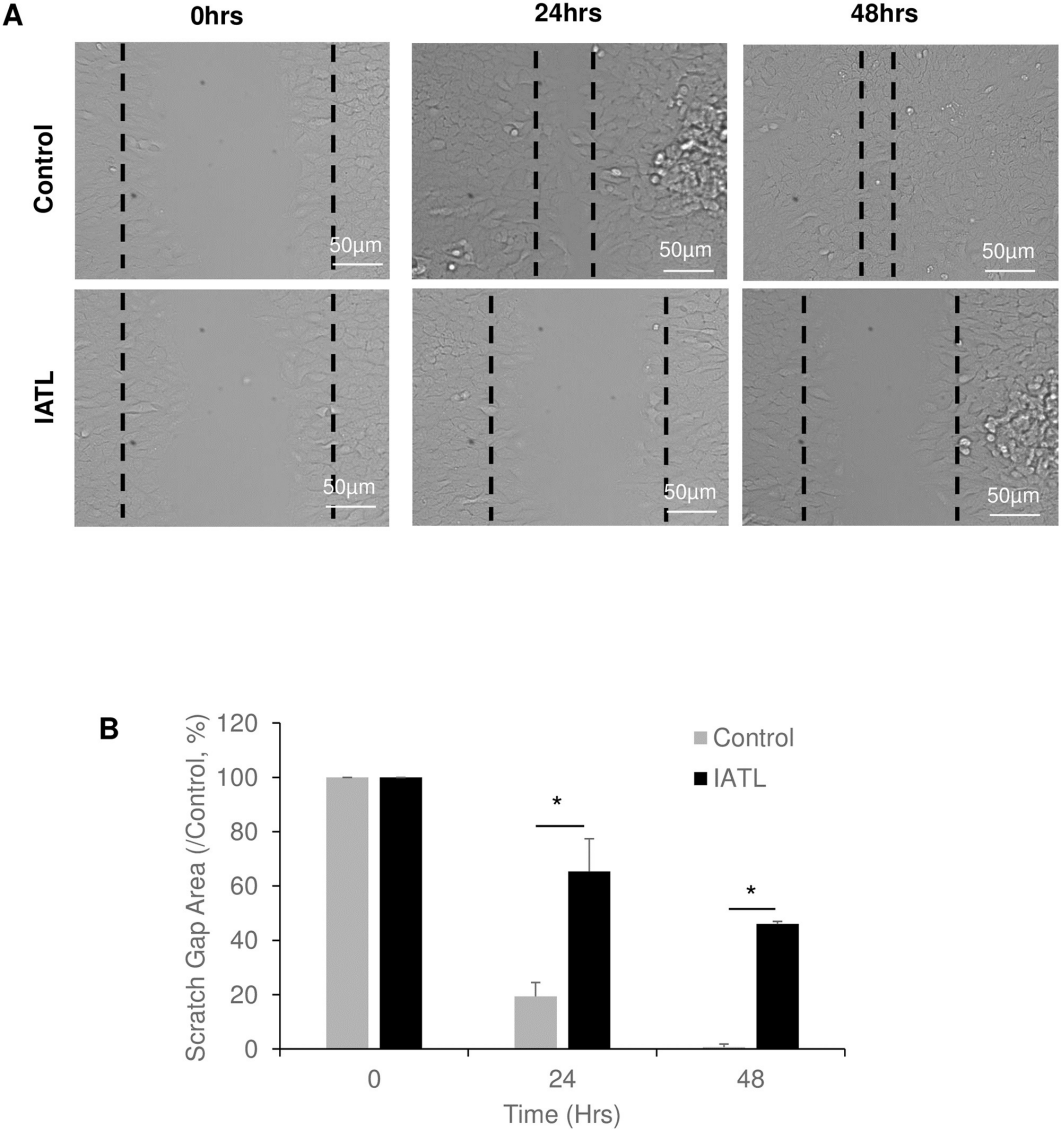
IATL inhibits pancreatic cancer cell migration. The confluent PANC-1 cells were scratched followed by treatment with 20 μM IATL at the indicated times. (A) Representative scratch images of PANC-1 in the presence or absence of IATL; (B) IATL significantly inhibited scratch closure of PANC-1 as compared to vehicle treatment. The PANC cell closure areas were represented as percentage of 0 hours, respectively (P<0.05, n = 3).

The cell scratch closure assay showed that the scratch gap was significantly closed in PANC-1s control group after 24 hours incubation and completely closed at 48 hours (Fig 2A). However, IATL-treated PANC-1s significantly delayed the gap closures at both 24hours and 48 hours incubations (Fig 2A and 2B; P<0.05). These results indicated that IATL-mediated PANC-1 toxic effects may include inhibition of cellular differentiation and migration.

### IATL inhibited pancreatic cancer cell invasion

**Fig 3:**
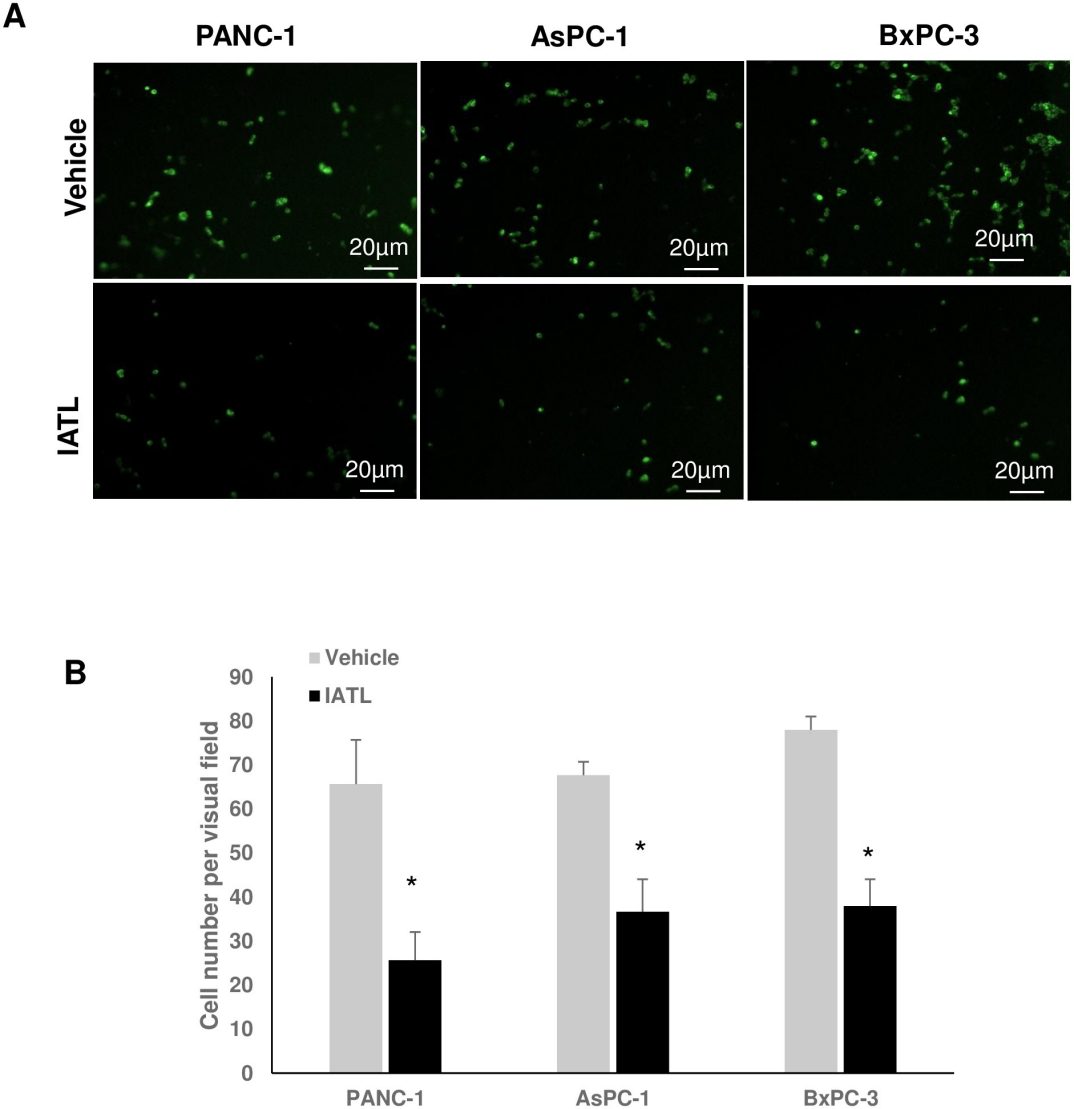
IATL inhibits pancreatic cancer cell invasion. The GFP-expressing PANC-1, AsPC-1 or BxPC-3 cells were seeded into transwell inserts, which were previously coated with matrigel. The PANC-1, AsPC-1 or BxPC-3 cells (1×10^5^) were treated with 20 μM IATL or vehicle for 24hours. The insert membranes were cut and mounted onto coverslides and the images were taken using fluorescent microscopy. (A) Representative cell images on the insert member after IATL or vehicle treatment for 24 hours; (B) Compared to vehicle, IATL treatment significantly inhibited invasion in all three different pancreatic cancer cell lines (P<0.05, n = 4).

PANC-1s, AsPC-1s or BxPC-3s (1×10^5^) expressed GFP by transfecting pLV-GFP virus. Cells were then cultured on matrigel coated transwell insert. After incubation for 16 hours, the GFP positive cells on the bottom side of the transwell insert member were counted using fluorescent microscopy (Fig 3A). In the control groups, the average number of invaded PANC-1s, AsPC-1s or BxPC-3s under an individual field were 65.67±10.1, 66.67±3.1, 78.0±3.1, respectively. However, 20μM IATL treatment for 16 hours significantly inhibited pancreatic cancer cell invasion. The average invaded number of PANC-1s, AsPC-1s or BxPC-3s under individual field were 25.67±6.4, 36.67±7.37, 38.0±6.08, respectively (Fig 3B; P<0.01).

### IATL inhibited AMPK-SKP2-Akt signal pathway in pancreatic cancer cells

**Fig 4:**
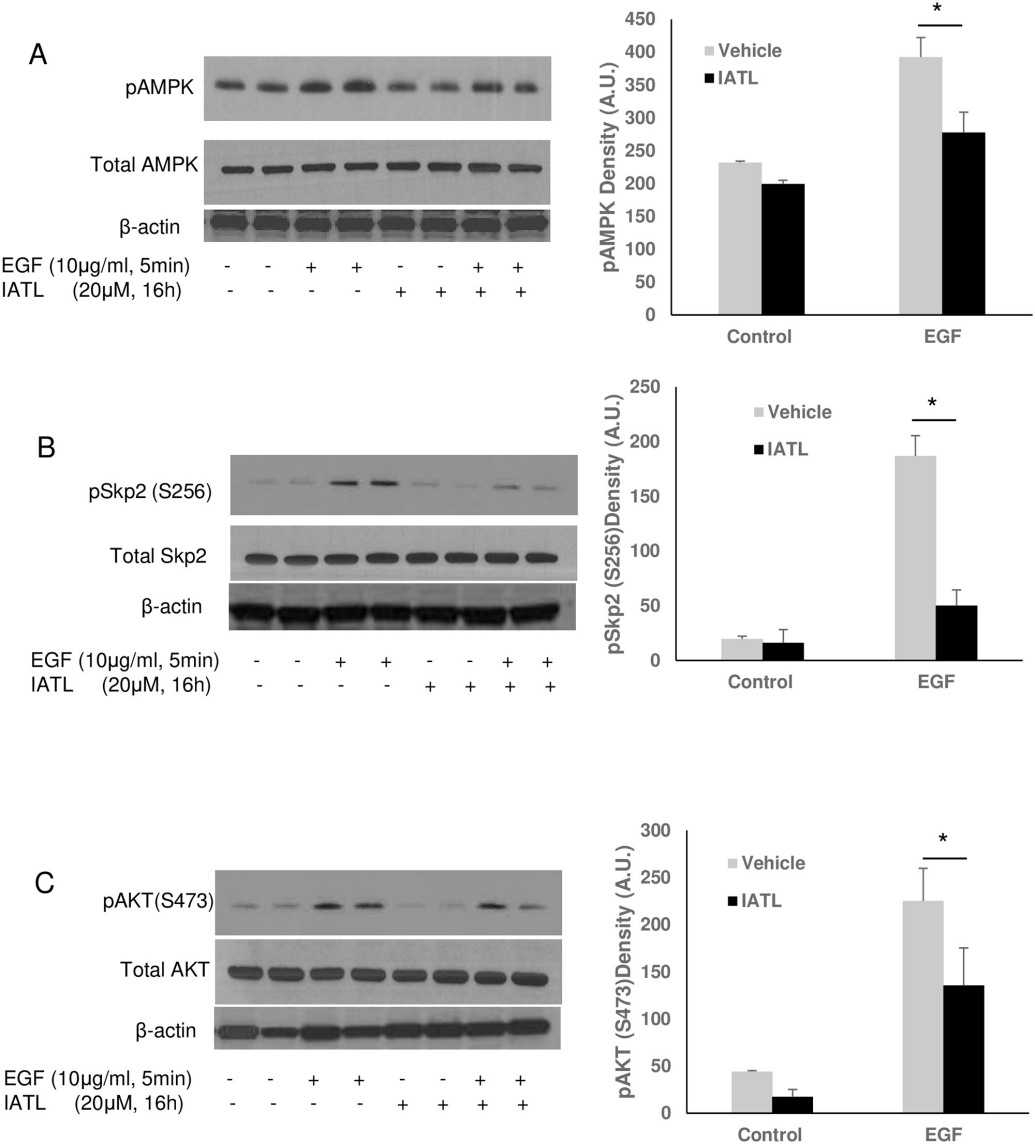
IATL inhibits EGF signal pathway through Skp2 inactivation in pancreatic cancer cells. The PANC-1 cells were treated with 20μM IATL followed by EGF (10μg/mL) treatment for 5minutes. After the cell lysates were harvested, the target protein phosphorylation was determined by western blotting. EGF caused AMPK (A), Skp2 (B) and Akt (C) phosphorylation increases; but IATL significantly inhibited EGF-mediated EGF (A)-SKp2 (B) and Akt (C) phosphorylation (P<0.05, n = 3).

Previous studies indicated that multiple signal pathways regulate pancreatic carcinoma cell apoptosis or proliferation, including PI3K-akt-mTOR signal pathway [23], AMPK-Akt signal pathway [24, 25] or Wnt signal pathway [26]. To investigate the detailed mechanism of IATL-mediated pancreatic carcinoma cell apoptosis, we first tested the effect of IATL on AMPK-Skp2-Akt signal pathway in pancreatic cancer cells. As shown in Fig 4A, EGF caused a significant phosphorylation of AMPK (Fig 4A), Skp2 (Fig 4B)- and Akt (Fig 4C) in PNAC-1s. However, EGF-mediated activation of AMPK, Skp2, and Akt were significantly inhibited by IATL (Fig 4).

### IATL inhibited canonical Wnt signal pathway in PANC-1

**Fig 5:**
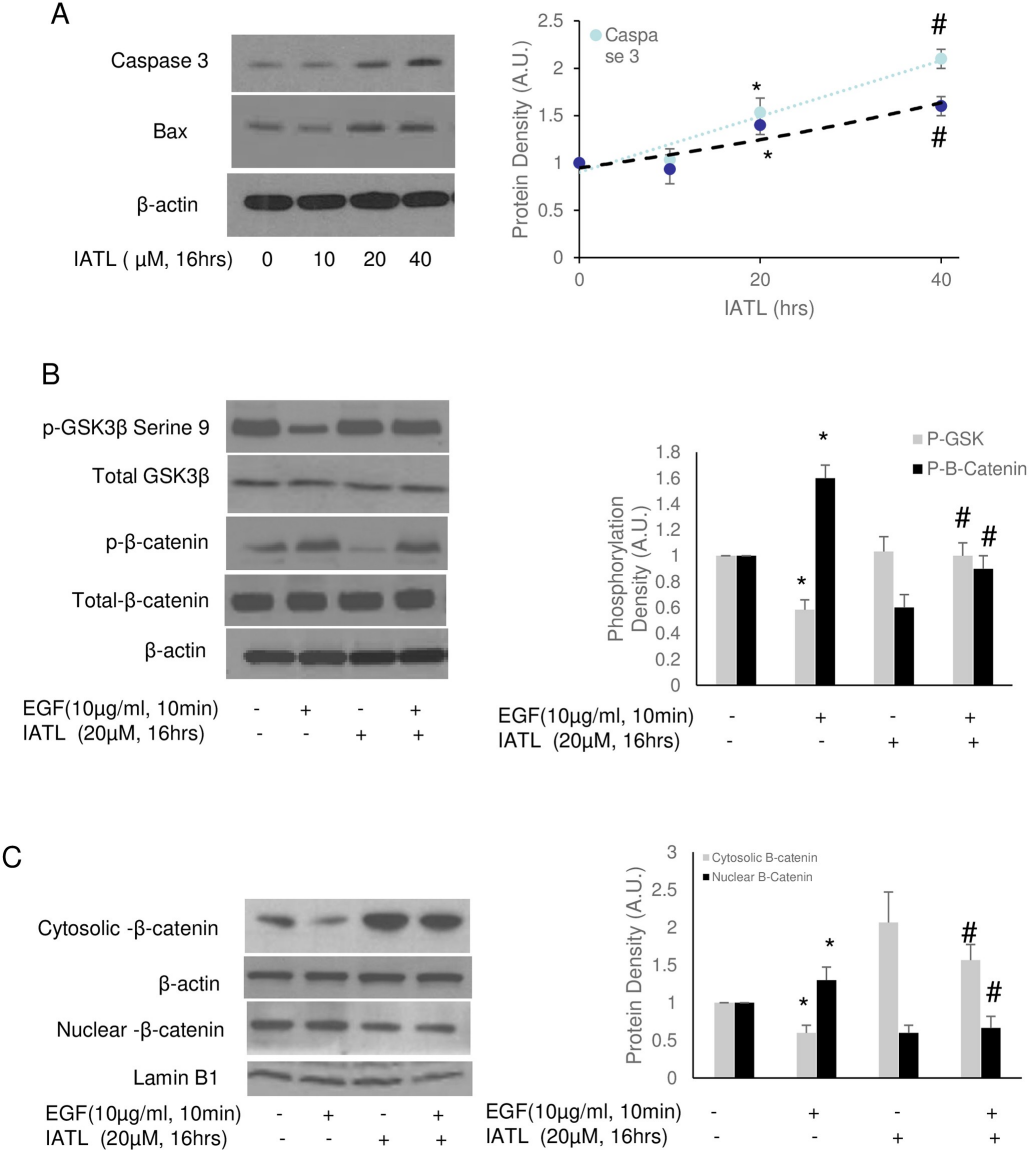
IATL inhibits Wnt signal pathway and induces pancreatic cancer cells apoptosis. The PANC-1 cells were treated with 20μM IATL alone or in combination with EGF (5μg/mL) at the indicated time. The cell lysates were harvested for western blotting. (A) IATL induced both Caspase 3 and Bax in PANC-1 cells in a dose-dependent fashion; (B) EGF activated the Wnt signal pathway by decreasing serine 9 phosphorylation of GSK-3β (*P<0.05, n = 3), however, IATL increased GSK-3β phosphorylation (#P<0.05, n = 3); EGF increased β-catenin phosphorylation (*P<0.05) but IATL reversed this regulation (#P<0.05, n = 3); (C) EGF caused β-catenin translocation into cellular nucleus (*P<0.05, n = 3) but IATL inhibited EGF-mediated β-catenin translocation (#P<0.05, n = 3).

By regulation of β-catenin, the Wnt signal pathway regulates the development of various cancers, such as breast, colorectal, melanoma, prostate cancer and pancreatic cancer [27]. The regulatory mechanisms of Wnt include cell proliferation, migration, and cell specification [28]. Therefore, we then investigated whether Wnt signal pathways regulated IATL-mediated pancreatic carcinoma cell apoptosis. Western blots showed that IATL induced release of capase-3 and Bax dose-dependently in PANC-1(Fig 5A). 20μM IATL was enough to induce PANC-1 apoptosis according to caspase-3 and Bax release. Serine 9 phosphorylation of GSK-3β, an inhibitory index of GSK was decreased by EGF but was reversed by IATL. In addition, IATL caused decrease in both basal and EGF-mediated β-catenin phosphorylation (Fig 5B). Interestingly, IATL decreased β-catenin accumulation in cellular nucleus (Fig 5C).

### IATL inhibited proliferation of pancreatic cancer cells xenograft *in vivo*

**Fig 6:**
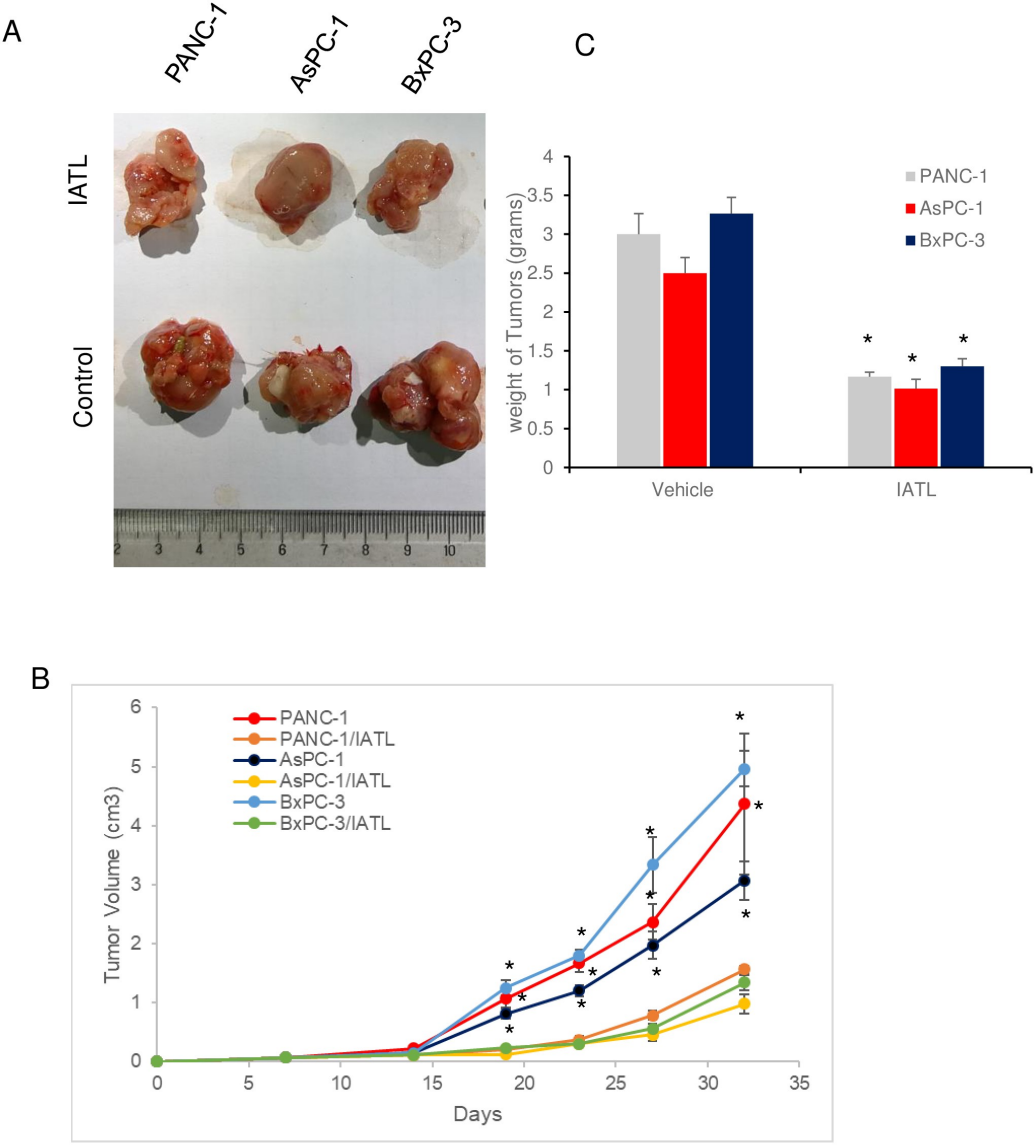
IATL inhibits pancreatic cancer cells growth *in vivo*. The PANC-1, AsPC-1 or BxPC-3 cells were subcutaneously xenografted onto BALB/c nude mice flank. Once the cells were injected, the mice were treated with IATL (0.5mg/kg) or vehicle once a week for 5 weeks. At the end of experiment, the xenografts were removed for assessing weight or for imaging. (A) Images of representative tumors from PANC-1, AsPC-1 or BxPC-3 cell xenografts (n = 6). (B) Growth curve of tumors xenografts in the presence or absence of IATL over 5 weeks; (C) IATL significantly decreased the weight of tumors generated from PANC-1, AsPC-1 or BxPC-3 cells *in vivo* (*, P<0.05).

The three different pancreatic cancer cell line, PANC-1s, AsPC-1s or BxPC-3s (1×10^6^) were injected into left frontier axilla subcutaneously for 5 weeks and the tumor growth was monitored at indicated time (Fig 6B). Once the cells were injected into mice, the mice were treated with IATL (0.5mg/kg, once/week) or vehicle for 5 weeks. Compared to the vehicle treatment group, IATL treatment significantly reduced the tumor sizes of PANC-1s xenografts (4.3±1.2 vs 1.3±0.07 cm^3^; vehicle vs ITAL, Fig 6A and 6B, P<0.05). Similar toxic effects were also observed with AsPC-1s (3.07±0.32vs 0.97±0.17 cm^3^; vehicle vs ITAL, Fig 6A and 6B, P<0.05) and BxPC-3s xenografts treated with IATL (4.96±0.30 vs 1.34±0.127 cm^3^; vehicle vs ITAL, Fig 6A and 6B, P<0.05).

Consistently, IATL also significantly decreased the tumor weight of PANC-1s xenografts (3.0±0.26 vs 1.16±0.06 g; vehicle vs ITAL, Fig 6C, P<0.05). Similar toxic effects were also observed with AsPC-1s (2.5±0.2 vs 1.01±0.12 g; vehicle vs ITAL, Fig 6C, P<0.05) and BxPC-3s xenografts treated with IATL (3.26±0.21 vs 1.29 ±0.10 g; vehicle vs ITAL, Fig 6C, P<0.05).

### IATL induced apoptosis of PANC-1 xenograft *in vivo*

**Fig 7:**
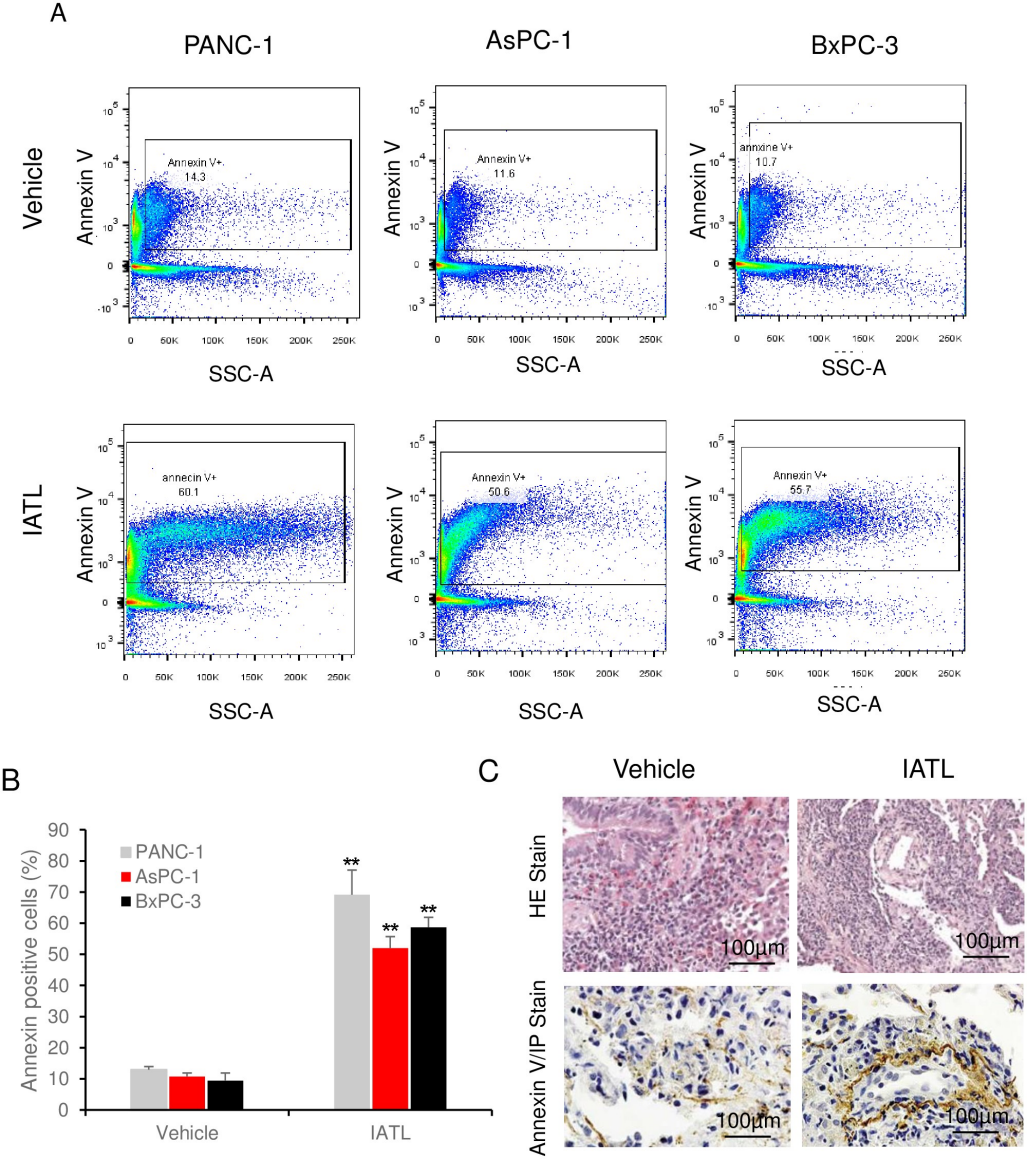
IATL induces pancreatic cancer cells apoptosis *in vivo*. The PANC-1, AsPC-1 or BxPC-3 cells were xenografted on BALB/c nude mice and were treated with IATL. In the end of experiment, the xenografts on nude mice were removed for flowcytometry or histology assay. (A) Representative images of flow cytometry assay showing detection of annexin-V positive cells from PANC-1, AsPC-1 or BxPC-3 xenografts for both vehicle and IATL treatment groups; (B) Compared to vehicle, IATL treatment significantly increased annexin-V positive cells in PANC-1, AsPC-1 or BxPC-3 cell xenografts (*, P<0.05; **, <0.01). (C) Representative images of H&E staining (upper panel) and annexin V staining (bottom panel) of PANC-1s xenografts (n = 6).

To further evaluate the effect of IATL on pancreatic cancer *in vivo*, flow cytometry was performed on the cells isolated from xenograft. Staining cells isolated from PANC-1 xenograft with Annexin V-Propidium iodide (PI) showed that IATL treatment caused a significantly higher annexin-V positive cell ratio comparing to vehicle groups (Fig 7A). The annexin-V positive cell ratio in PANC-1 xenograft were 13.0 ±1.1 vs 69.13±7.94%; vehicle vs ITAL (Fig 7B, P<0.05). A similar toxic effect of IATL against AsPC-1s (10.78±1.15 vs 52.3±3.7%; vehicle vs ITAL, Fig 7B, P<0.05) and BxPC-3s (9.4±2.49 vs 58.67±3.22%; vehicle vs ITAL, Fig 7B, P<0.05) in respective xenografts was also observed.

Moreover, H&E stain and immunohistological assays were performed after PANC-1 xenografts were isolated from mice. The samples from both vehicles and IATL treatment groups showed typical carcinoma characteristics, such as lower level organized tissue, poor blood vessel development and dark stain of cellular nucleus (Fig 7C upper panel). However, annexin-V stain showed that the annexin-V positive cell number in IATL groups was significantly higher than those in vehicle groups (P<0.05, Fig 7C, bottom panel).

## Discussion

### Discussion

IATL demonstrated significant inhibitory effects on pancreatic carcinoma cell proliferation, migration and invasion. Xenograft studies also showed that IATL significantly inhibited pancreatic carcinoma cell transplants growth *in vivo*. Signal pathway studies demonstrated that IATL induces pancreatic carcinoma cell apoptosis by inhibition of the non-canonical wnt signal pathway. IATL inhibits pancreatic carcinoma cells proliferation by inhibition of AMPK-Skp2-Akt signal pathway. S-phase kinase associated protein 2 (Skp2) is a member of the F-box family, which regulates the cell cycle and is highly expressed in various carcinomas, such as pancreatic [29], breast [24] and kidney carcinoma [30]. Skp2 recruits Skp1, cullin-1 and Rbx1E3 ligase to form a SCFSkp2 complex, which targets cell cycle elements, such as p27, p21 and further regulates cell cycle entry and G1/S transition [31–33]. Silence of Skp2 or inhibition Skp2 by pharmaceutic inhibitors significantly improves breast cancer cell sensitivity to the anti-breast cancer drug, Gefitinib. Further studies indicate that Skp2 plays a central role in EGF-mediated signal activation, such as phosphorylation and ubiquitination of Akt. Akt ubiquitination further causes tumorigenesis and drug resistance [24]. In the current study, IATL significantly inhibits EGF-mediated Skp2 serine phosphorylation. Moreover, AMPK-Skp2-Akt signal pathway appears to be involved in IATL-mediated effects as both AMPK and Akt were dephosphorylated as Skp2 was simultaneously dephosphorylated. These results are consistent with previous observations in different models [24, 34].

The Wnt signal pathway is another signal pathway regulating many cell fates, including apoptosis, cell death and inflammatory responses. The Wnt signal pathway includes non-canonical and canonical Wnt signal pathways [35]. Cadmium inhibited the canonical wnt signal pathway, which in turn induces non-small cell lung carcinoma cell apoptosis [26]. Early gene profiling studies indicate that Wnt5a, a marker of the non-canonical wnt molecule is up-regulated in pancreatic ductal adenocarcinoma cells [36]. DKK1 is also involved in pancreatic ductal carcinoma and is a potential biomarker [37]. Wnt inhibitors significantly reduce human pancreatic carcinoma cell viability in cellular and *in vivo* model [38]. Frizzled family receptor 8 inhibitor, OMP-54F28 inhibits advanced solid tumor cells, including pancreatic carcinoma cell proliferation [39]. In the current study, we showed that IATL inhibited the canonical wnt signal pathway in pancreatic carcinoma cells and induced apoptosis.

PRI-724 is the specific Wnt pathway inhibitor, which binds to the coactvator CBP, inhibiting its interaction with β-catenin [40]. Here we showed that IATL deceased β-catenin phosphorylation and β-catenin accumulation in cellular nucleus. The detail of molecular interaction need further investigation.

As a most commonly diagnosed cancer worldwide, anti-pancreatic carcinoma medicines and their mechanisms have been intensively investigated. By inhibiting DNA damage repairing mechanism, poly (ADP-ribose) polymerase inhibitors caused accumulation of double stranded DNA breaks and induced pancreatic ductal adenocarcinoma cell death [41]. By inhibiting some of the specific signal pathways, such as the Src signal pathway or the KRas signal pathway, these medicines also appear to work efficiently on pancreatic carcinoma [42, 43]. The manipulation of ROS as in the drug-mediated apoptosis in pancreatic carcinoma has also been widely investigated [44]. However, ROS-induced apoptosis in pancreatic carcinoma was mediated by specific target pathway or down-stream target transfactors or proteins [45, 46]. Previous reports have also shown that ROS functions as a mediator in IATL-induced toxic effects of pancreatic carcinoma [14, 47]. Meanwhile, the studies also showed that specific signal pathways, such as STAT3, MAPK may also play critical roles in the process of apoptosis [14, 47]. However, the regulatory relationship between ROS and these signal pathways remain unclear. To our knowledge, the current study characterized for the first time the involvement of the EGF- AMPK-Skp2-Akt signal pathway and canonical Wnt signal pathway in IATL-mediated apoptosis of pancreatic carcinoma. Considering the importance of EGF- AMPK-Skp2-Akt signal pathway in tumorigenesis, metastasis and invasion, IATL may provide a promising medicine for pancreatic cancer. Moreover, Skp2 is a central signal protein related to both EGF and drug resistance [29, 48]. The inhibitory effect of IATL on both EGF and SKP2 may offer a novel option for pancreatic cancer.

## Conclusion

In summary, our present studies indicate that IATL inhibits pancreatic carcinoma by inhibition of the EGF- AMPK-Skp2-Akt signal pathway. IATL induces pancreatic carcinoma apoptosis by inhibition of the canonical wnt signal pathway. Moreover, IATL shows a significant inhibitory effect of pancreatic carcinoma in an *in vivo* xenograft model. These studies of IATL on pancreatic cancer may offer opportunities for both pharmaceutic and clinical purposes in the future.

## Supporting information

S1 Raw images (PDF)
