# Supplementary material for: Isoalantolactone inhibits pancreatic cancer proliferation by regulation of PI3K and Wnt signal pathway
Source: PLoS One. 2021 Mar 4;16(3):e0247752. doi: 10.1371/journal.pone.0247752 (PMC7932101; doi:10.1371/journal.pone.0247752)

11/9/17

Figure 4B

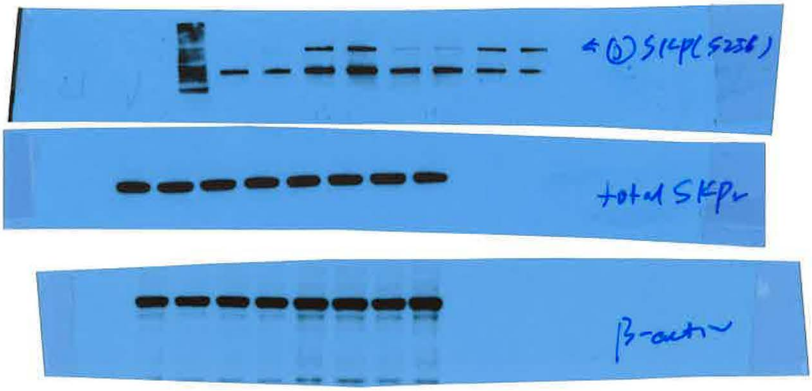

11/20/17

Figure 4C

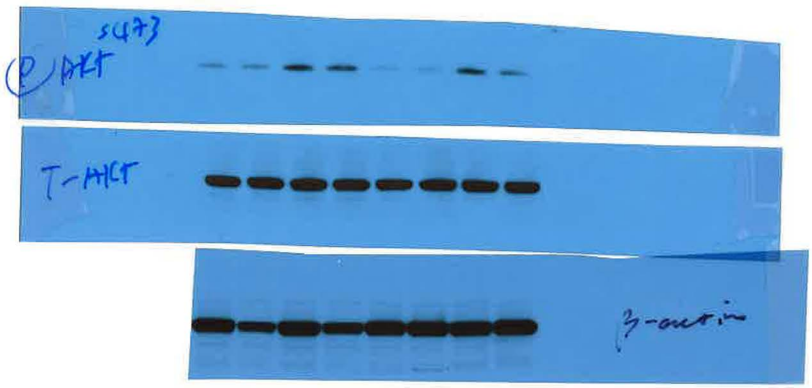

03/31/2015

Figure 5A

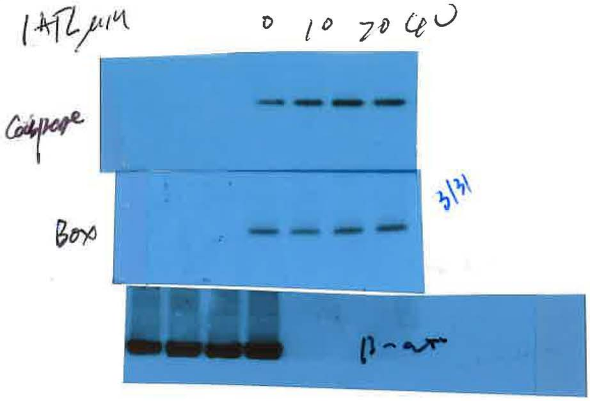


Figure 5B

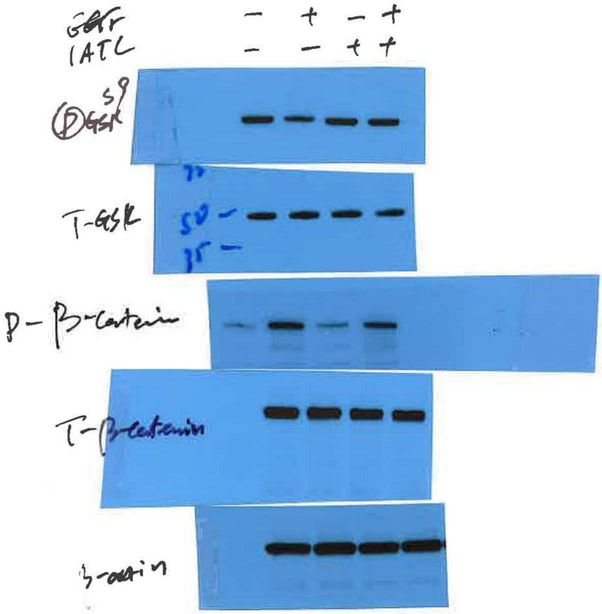

07/13/15

Figure 5 C

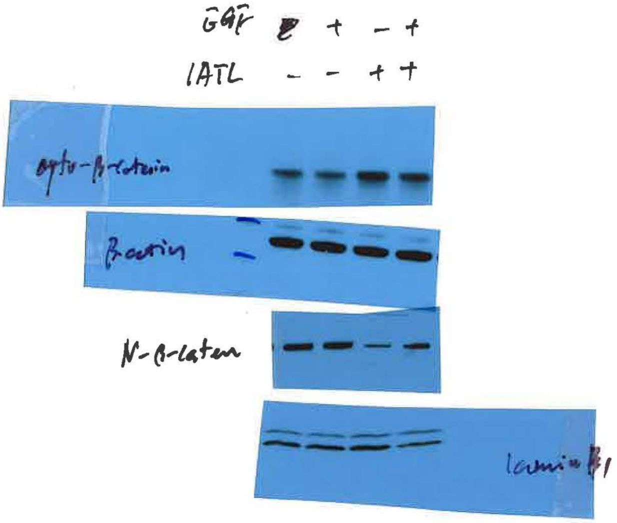

11/09/17

Figure 4A

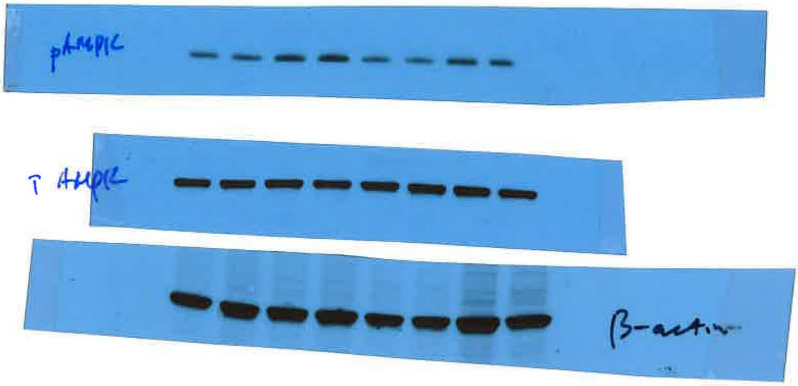

Supplement: S1 Raw images — (PDF) [file pone.0247752.s001.pdf]
